# Sorafenib Treatment and Modulation of the Sphingolipid Pathway Affect Proliferation and Viability of Hepatocellular Carcinoma In Vitro

**DOI:** 10.3390/ijms21072409

**Published:** 2020-03-31

**Authors:** Katja Jakobi, Sandra Beyer, Alexander Koch, Dominique Thomas, Stephanie Schwalm, Stefan Zeuzem, Josef Pfeilschifter, Georgios Grammatikos

**Affiliations:** 1 Medizinische Klinik 1, University Hospital, Goethe University Frankfurt am Main, 60590 Frankfurt, Germany; jakobi@med.uni-frankfurt.de (K.J.); zeuzem@em.uni-frankfurt.de (S.Z.); 2 Institut für Allgemeine Pharmakologie und Toxikologie, University Hospital, Goethe University Frankfurt am Main, 60590 Frankfurt, Germany; S.Beyer@med.uni-frankfurt.de (S.B.); Koch@med.uni-frankfurt.de (A.K.); s.schwalm@med.uni-frankfurt.de (S.S.); Pfeilschifter@em.uni-frankfurt.de (J.P.); 3 Institut für Klinische Pharmakologie und Toxikologie, University Hospital, Goethe University Frankfurt am Main, 60590 Frankfurt, Germany; Thomas@med.uni-frankfurt.de; 4 St Luke’s Hospital, 55236 Thessaloniki, Greece

**Keywords:** liver, HCC, dihydroceramide, SKI II, fumonisin B1

## Abstract

Hepatocellular carcinoma (HCC) shows a remarkable heterogeneity and is recognized as a chemoresistant tumor with dismal prognosis. In previous studies, we observed significant alterations in the serum sphingolipids of patients with HCC. This study aimed to investigate the in vitro effects of sorafenib, which is the most widely used systemic HCC medication, on the sphingolipid pathway as well as the effects of inhibiting the sphingolipid pathway in HCC. Huh7.5 and HepG2 cells were stimulated with sorafenib, and inhibitors of the sphingolipid pathway and cell proliferation, viability, and concentrations of bioactive metabolites were assessed. We observed a significant downregulation of cell proliferation and viability and a simultaneous upregulation of dihydroceramides upon sorafenib stimulation. Interestingly, fumonisin B1 (FB1) and the general sphingosine kinase inhibitor SKI II were able to inhibit cell proliferation more prominently in HepG2 and Huh7.5 cells, whereas there were no consistent effects on the formation of dihydroceramides, thus implying an involvement of distinct metabolic pathways. In conclusion, our study demonstrates a significant downregulation of HCC proliferation upon sorafenib, FB1, and SKI II treatment, whereas it seems they exert antiproliferative effects independently from sphingolipids. Certainly, further data would be required to elucidate the potential of FB1 and SKI II as putative novel therapeutic targets in HCC.

## 1. Introduction

Hepatocellular carcinoma (HCC) is recognized worldwide as a devastating malignancy since it constitutes the second-leading cause of cancer-related mortality, particularly in men [1] and shows a remarkable resistance profile against systemic therapies [2]. In contrast to the optimization of surgical and locoregional therapies driven by the improvement of surveillance of patients at risk, the only available systemic option for the last 10 years was restricted to sorafenib, which is a multi-kinase inhibitor with limited efficacy in patients with unresectable HCC [3]. Recently, further medications have been approved as additional first-line or second-line treatments of HCC, yet they merely show just a non-inferiority as compared to sorafenib [2]. Collectively, HCC is still broadly recognized as an aggressive and chemoresistant tumour with the efficacy of systemic HCC treatment remaining disappointingly low.

Sphingolipids are bioactive lipid molecules containing a sphingoid backbone attached to a fatty acid of variable chain length and composition, and they have gained remarkable attention in oncologic therapeutic approaches. The functional sphingolipid rheostat between ceramide and sphingosine 1-phosphate (S1P) has been well characterized in cancer therapy [4], and sphingolipids are playing a central role in the pathophysiology of various tumors [5]. Especially in hepatic homeostasis, several studies observed an implication of (dihydro-) ceramides and S1P as major regulators of hepatocellular susceptibility to various stimuli [6,7] as well as of hepatocarcinogenesis both in vitro and in vivo [7,8,9,10,11,12]. Furthermore, modification of the sphingolipid pathway by the inhibition of regulating enzymes has been shown to play a significant role in the pathogenesis of tumors [13,14]. Particularly, previous observations have revealed an additive effect of the selective sphingosine kinase 2 (SPHK2) inhibitor ABC294640 together with sorafenib on cell toxicity and death in HCC cells in vitro and the increased suppression of tumor growth in murine HCC models [15]. In the same context and with adequate oral bioavailability, the dual sphingosine kinase inhibitor SKI II has been shown to act synergistically to fluorouracil in suppressing the proliferation and viability of HCC in vitro [16]. The exogenous addition of acid sphingomyelinase or ceramide has been shown to augment the anti-tumor efficacy of sorafenib [17,18]. Further data showed an upregulation of ceramide-induced cell death upon the stimulation of tumor cells with sorafenib and vorinostat [19,20] and a reversion of chemoresistance to sorafenib by the targeting of glucosylceramide synthase [21]. In addition, ceramide synthases inhibitor fumonisin B1 (FB1) has been linked with a regulatory role in HCC pathophysiology [22,23].

However, it remained largely unknown whether the antiproliferative effects of sorafenib imply an involvement of the sphingolipid pathway. Recently published studies from our group and by others revealed significant alterations of the serologic sphingolipid profile in patients with chronic liver disease and especially with HCC [24,25,26] partially also predicting the effectiveness of sorafenib treatment [27]. The significant upregulation of ceramides and dihydroceramides observed in the serum of HCC patients raises the question if responsiveness to anti-HCC treatment may be affected or even get ameliorated by alterations of these metabolic compounds.

Thus, the purpose of this study was to investigate whether stimulation with sorafenib, FB1, and SKI II affects the sphingolipid metabolism in a mechanistic way and whether modulation of the sphingolipid pathway is able to affect the proliferation and viability of in vitro models of HCC. The effects of the selective sphingosine kinase 1 (SPHK1) inhibitor SLP7111228 (SLP) and the selective SPHK2 inhibitor SLM6031434 (SLM) were therefore also investigated in this work (Figure 11). To the best of our knowledge, this is the first time the influence of sorafenib on sphingolipid formation was investigated in HCC cell lines in detail. In addition, the influence of sorafenib in combination with the specific sphingosine kinase (SPHK) inhibitors SLP and SLM was not investigated before.

## 2. Results

### 2.1. Effects of Sorafenib, FB1, and SPHK Inhibitors on Cell Proliferation

**Figure 1:**
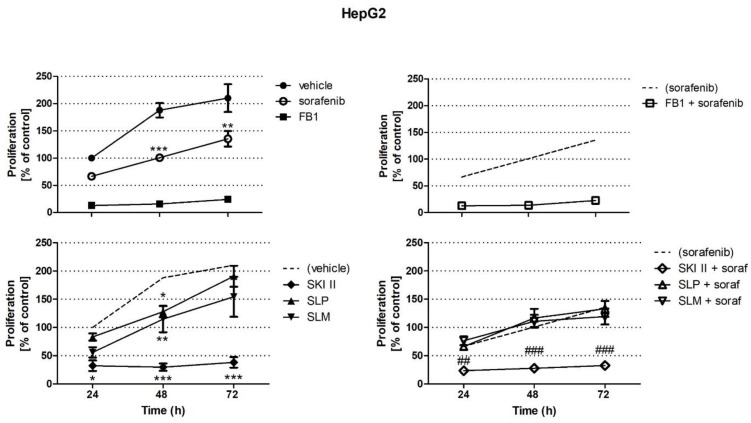
Influence of sorafenib, fumonisin B1 (FB1), and sphingosine kinase (SPHK) inhibitors on the proliferation of HepG2 cells. Cell proliferation was analyzed by measuring the incorporation of radioactively labeled thymidine. The cells were pretreated with 25 µM of FB1 for 1 h, or with 10 µM SKI II, 1 µM SLP, or 1 µM SLM for 2 h. Then, vehicle (control; 0.2 % DMSO) or 5 µM sorafenib (soraf) were added for the indicated periods of time. All data are derived from three independent experiments performed in triplicate, which comprised all conditions; FB1 was included in two of these experiments. The dashed lines repeatedly indicate vehicle control or sorafenib as shown in the upper left panel. The values are means ± SEM and expressed relative to vehicle control at 24 h. * *p* < 0.05, ** *p* < 0.01, *** *p* < 0.001 compared to control; ^##^ *p* < 0.01, ^###^ *p* < 0.001 compared to sorafenib in two-way ANOVA (*n* = 2 for FB1 and *n* = 3 for SK I II, selective SPHK2 inhibitor SLM6031434 (SLM), and SPHK1 inhibitor SLP7111228 (SLP)).

Since sorafenib is known to induce apoptosis in HCC, we investigated the influence of sorafenib and of FB1 and SPHK inhibitors on the proliferation of HepG2 and Huh7.5 cells. Therefore, we measured the incorporation of radioactively labeled thymidine after 24 h, 48 h, and 72 h of incubation. HepG2 and Huh7.5 cells were treated for 1 h with FB1, which is an inhibitor of ceramide synthases, before the addition of vehicle or 5 µM of sorafenib. Pretreatment with SPHK inhibitors was performed for 2 h before the addition of vehicle or sorafenib. As shown in Figure 1, the proliferation of HepG2 cells was significantly reduced by sorafenib after 48 h and 72 h of incubation. Furthermore, FB1 and the non-specific and non-selective SPHK inhibitor, SKI II, strongly reduced the proliferation of HepG2 cells at all time points. The inhibitory effects of FB1 and SKI II were much more pronounced than that of sorafenib (Figure 1). In contrast, the influence of the specific inhibitors of SPHK1 and SPHK2, SLP and SLM, was only minor. Treatment of HepG2 cells with SLP or SLM caused a significant reduction of cell proliferation solely after 48 h of incubation (Figure 1). Furthermore, it was investigated whether the effect of sorafenib could be enhanced by simultaneous treatment with FB1 or SPHK inhibitors. As shown in Figure 1, there is no difference between stimulation with SKI II or FB1 alone or in combination with sorafenib. In contrast, neither SLP nor SLM had an influence on sorafenib-induced reduction of HepG2 cell growth (Figure 1).

**Figure 2:**
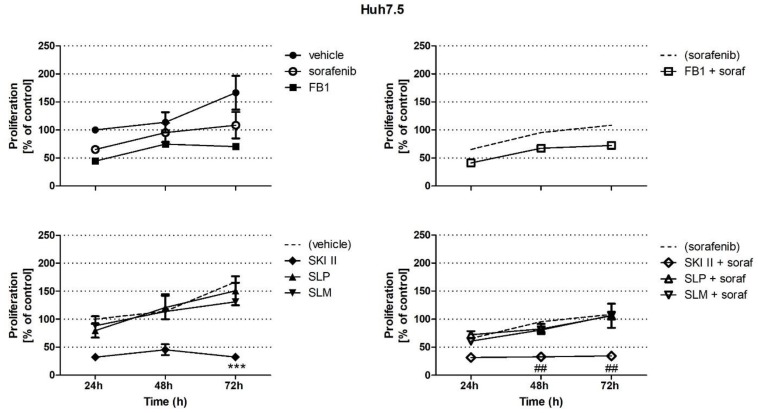
Influence of sorafenib, FB1, and SPHK inhibitors on the proliferation of Huh7.5 cells. Cell proliferation was analyzed by measuring the incorporation of radioactively labeled thymidine. The cells were pretreated with 25 µM FB1 for 1 h, or with 10 µM SKI II, 1 µM SLP, or 1 µM SLM for 2 h. Then, vehicle (control; 0.2 % DMSO) or 5 µM sorafenib (soraf) were added for the indicated periods of time. All data are derived from three independent experiments performed in triplicate, which comprised all conditions; FB1 was included in two of these experiments. The dashed lines repeatedly indicate vehicle control or sorafenib as shown in the upper left panel. The values are means ± SEM and expressed relative to vehicle control at 24 h. *** *p* < 0.001 compared to control; ^##^ *p* < 0.01 compared to sorafenib in Two-way ANOVA.

In contrast to HepG2 cells, the proliferation of Huh7.5 cells was not significantly reduced by sorafenib (Figure 2). In contrast to HepG2 cells, SKI II and FB1 inhibited Huh7.5 cell proliferation after 72 h of incubation (Figure 2). SKI-II together with sorafenib reduced cell proliferation as strongly as SKI-II alone (Figure 2). The specific SPHK1 and SPHK2 inhibitors, SLP and SLM, had no effect on Huh7.5 cell proliferation, neither alone nor in combination with sorafenib (Figure 2).

### 2.2. Influence of Sorafenib, FB1, and SKI II on Apoptosis and Necrosis

As shown in Figure 1, FB1 and SKI II induced a reduction in the proliferation of HepG2 cells. Furthermore, stimulation with SKI II also led to a reduction in the proliferation of Huh7.5 cells (Figure 2). A DNA fragmentation ELISA was used in order to distinguish whether stimulation of the cells with the distinct compounds leads to increased apoptosis or necrosis.

**Figure 3:**
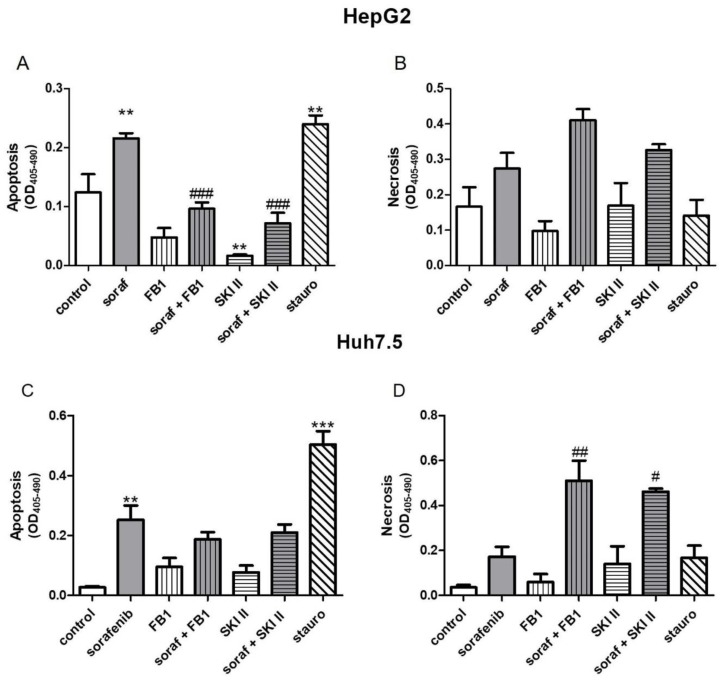
Influence of sorafenib, FB1, and S*KI II* on the apoptosis and necrosis of HepG2 and Huh7.5 cells. Apoptosis and necrosis were analyzed by measuring DNA fragmentation of the cytoplasmic fraction or in the supernatant. The cells (HepG2 in (**A**) and (**B**) and Huh 7.5 in (**C**) and (**D**)) were pretreated with 25 µM FB1 for 1 h, or with 10 µM SKI II for 2 h. Then, vehicle (control; 0.2% DMSO) or 5 µM sorafenib (soraf) were added for the indicated periods of time. Staurosporine (stauro) was used as a positive control. All data are derived from three independent experiments performed in triplicate. The values are means ± SEM and expressed relative to vehicle control at 24 h. ** *p* < 0.01, *** *p* < 0.001 compared to control; ^#^ *p* < 0.05, ^##^ *p* < 0.01, ^###^ *p* < 0.001 compared to sorafenib in a one-way analysis of variance with subsequent Bonferroni test. (*n* = 3 for all conditions).

As shown in Figure 3A,C, sorafenib and staurosporine as positive controls induced a significant increase in apoptosis in both cell lines compared to the vehicle control (0.2% DMSO). In HepG2 cells, stimulation with SKI II resulted in a significant reduction of apoptosis compared to the control (Figure 3A). Compared to sorafenib stimulation, the combination of sorafenib and FB1 or of sorafenib and SKI II resulted in reduced apoptosis (Figure 3A). None of the stimulations had an effect on the necrosis of HepG2 cells (Figure 3B). In the Huh7.5 cells, neither FB1 nor SKI II alone or in combination with sorafenib could affect the apoptosis of the stimulated cells (Figure 3C). As also shown in Figure 3D, in Huh7.5 cells, the combination of FB1 and sorafenib as well as of SKI II and sorafenib led to a significant increase in necrosis compared to the sole sorafenib stimulation.

### 2.3. Influence of Sorafenib, FB1, and SKI II on Levels of Bioactive Sphingolipids

**Figure 4:**
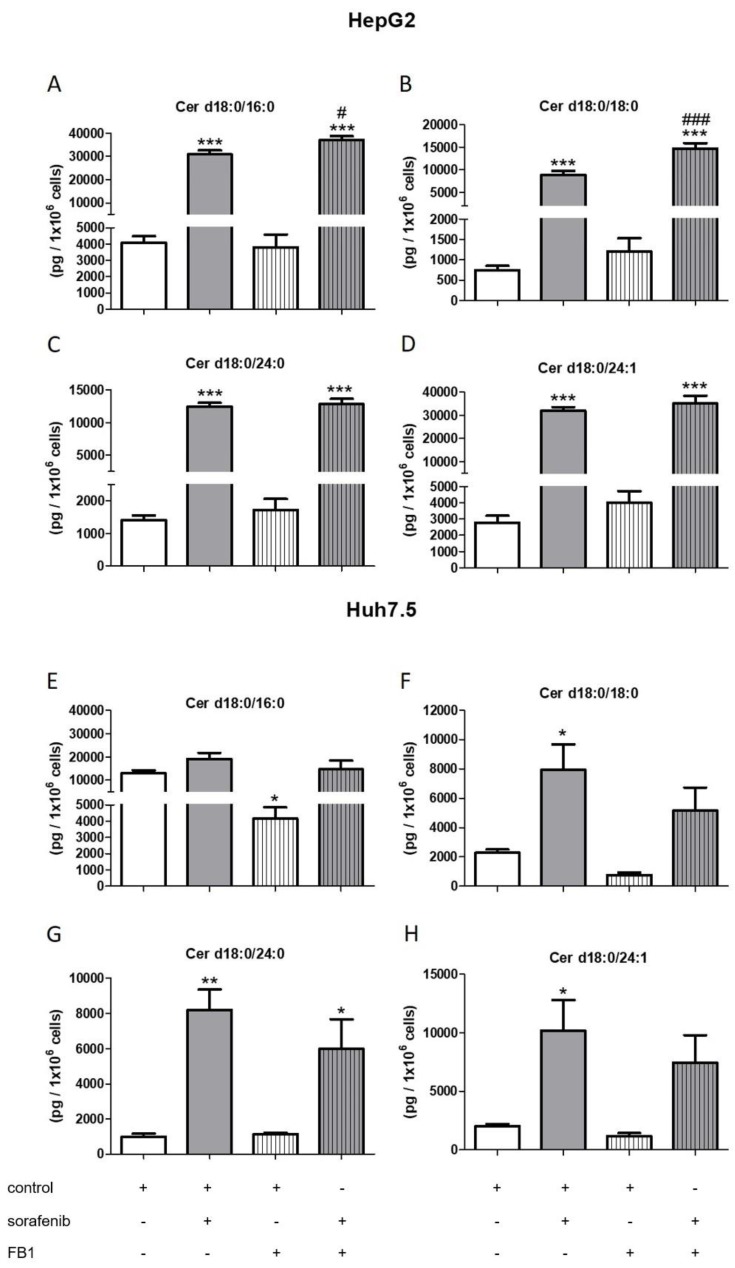
Influence of sorafenib and FB1 on concentrations of dihydroceramides in HepG2 and Huh7.5 cells. The lipids were measured by LC-MS/MS. The cells (HepG2 in (**A**)–(**D**) and Huh7.5 in (**E**)–(**H**)) were treated with or without 25 µM FB1 for 1 h before addition of vehicle (control; 0.2% DMSO) or 5 µM sorafenib and further incubation for 24 h. All results are presented as the means ± SEM of 3–6 independent experiments. * *p* < 0.05, ** *p* < 0.01, *** *p* < 0.001 compared to control; ^#^ *p* < 0.05, ^##^ *p* < 0.01, ^###^ *p* < 0.001 compared to sorafenib in one-way ANOVA followed by Bonferroni post-tests.

**Figure 5:**
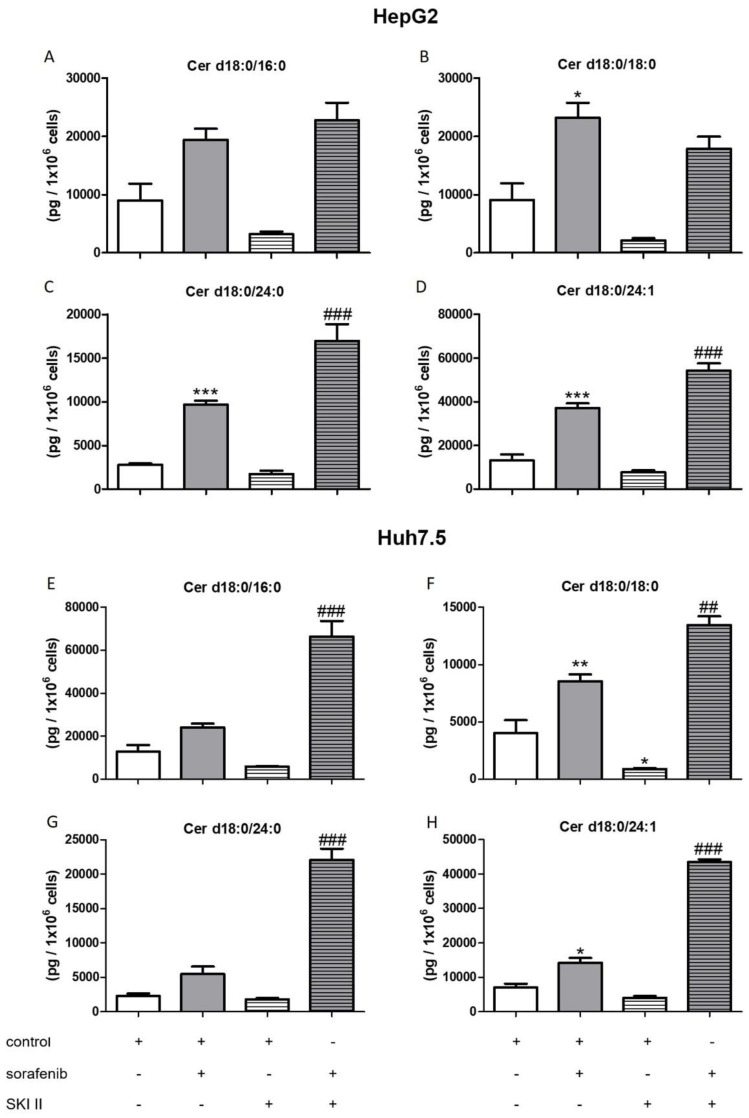
Influence of sorafenib and SKI II inhibitors on concentrations of dihydroceramides in HepG2 and Huh7.5 cells. The lipids were measured by LC-MS/MS. The cells (HepG2 in (**A**)–(**D**) and Huh7.5 in (**E**)–(**H**)) were treated with or without 10 µM SKI II for 2 h before the addition of vehicle (control; 0.2% DMSO) or 5 µM sorafenib and further incubation for 24 h. All results are presented as means ± SEM of three independent experiments. * *p* < 0.05, ** *p* < 0.01, *** *p* < 0.001 compared to control; ^##^ *p* < 0.01, ^###^ *p* < 0.001 compared to sorafenib in one-way ANOVA followed by Bonferroni post-tests.

In order to investigate if there is a connection between the observed reduced proliferation (Figure 1 and Figure 2), the induction of apoptosis (Figure 3A,C), and the alteration of the sphingolipid metabolism, we assessed concentrations of bioactive sphingolipid metabolites. Cells were pretreated with FB1 for 1 h before sorafenib or a vehicle were added. In both cell lines, sorafenib induced a strong increase in dihydroceramide concentrations, in particular, of Cer d18:0/18:0, Cer d18:0/24:0, and Cer d18:0/24:1 (Figure 4 and Figure 5). Interestingly, the inhibitor of ceramide synthases, FB1, did not diminish dihydroceramide concentrations in both cell lines, except from Cer d18:0/16:0 in Huh7.5 cells. On the contrary, FB1 further increased sorafenib-induced elevations of Cer d18:0/16:0 and Cer d18:0/18:0 in HepG2 cells (Figure 4A–D). In Huh7.5 cells, FB1 had no significant influence on sorafenib-induced elevations of dihydroceramides (Figure 4E–H).

Furthermore, the influence of the SPHK inhibitors, SKI II, SLP, and SLM on sphingolipid metabolites was measured. Cells were pretreated with SKI II, SLP, or SLM for 2 h before sorafenib or vehicle were added. None of the inhibitors altered dihydroceramides levels in both cell lines when administered alone (Figure 5). Again, co-treatment with SLP and SLM did not alter sorafenib-induced increases in dihydroceramide levels in the two cell lines (Table S1).

Interestingly, co-treatment of HepG2 cells with SKI II further elevated sorafenib-induced increase in the dihydroceramides, Cer d18:0/24:0 and Cer d18:0/24:1 (Figure 5C,D). In Huh7.5 cells, SKI II strongly augmented the effects of sorafenib on all measured dihydroceramides (Figure 5E–H).

**Figure 6:**
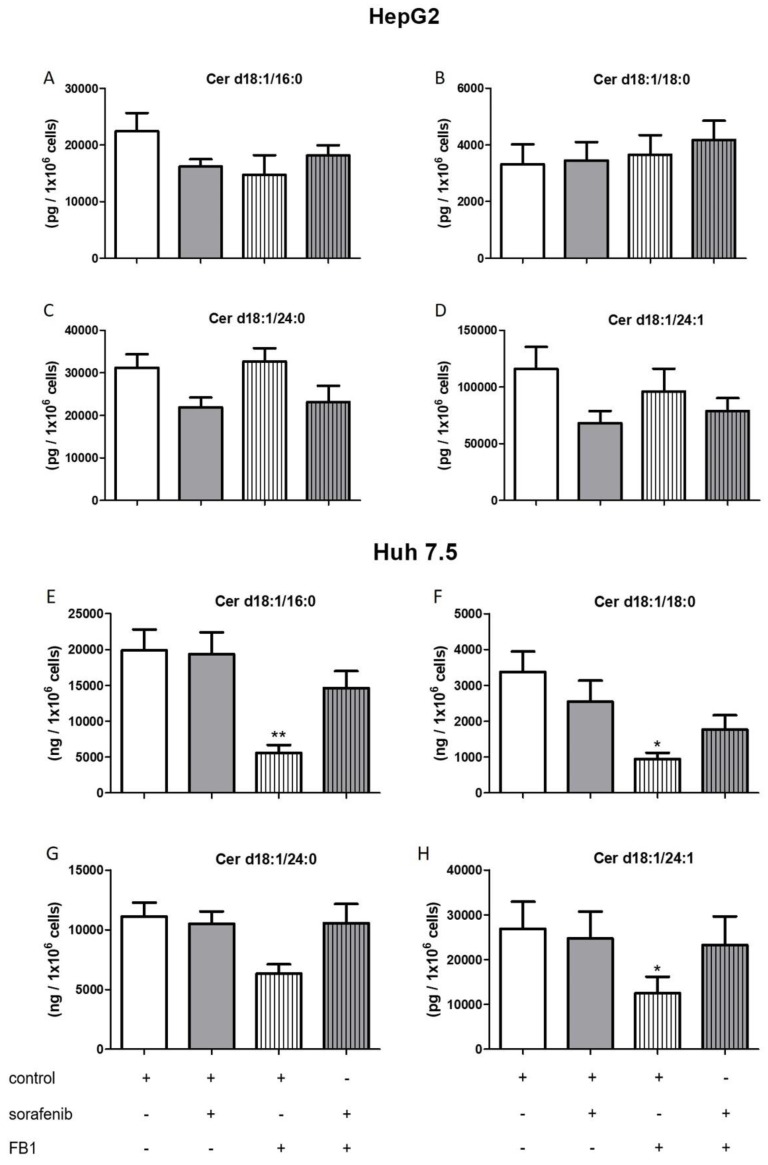
Influence of sorafenib and FB1 on concentrations of d18:1 ceramides in HepG2 and Huh7.5 cells. The lipids were measured by LC-MS/MS. The cells (HepG2 in (**A**)–(**D**) and Huh7.5 in (**E**)–(**H**)) were treated with or without 25 µM FB1 for 1 h before the addition of vehicle (control; 0.2% DMSO) or 5 µM sorafenib and further incubation for 24 h. All results are presented as means ± SEM of 3–6 independent experiments. * *p* < 0.05, ** *p* < 0.01 compared to control in one-way ANOVA followed by Bonferroni post-tests.

**Figure 7:**
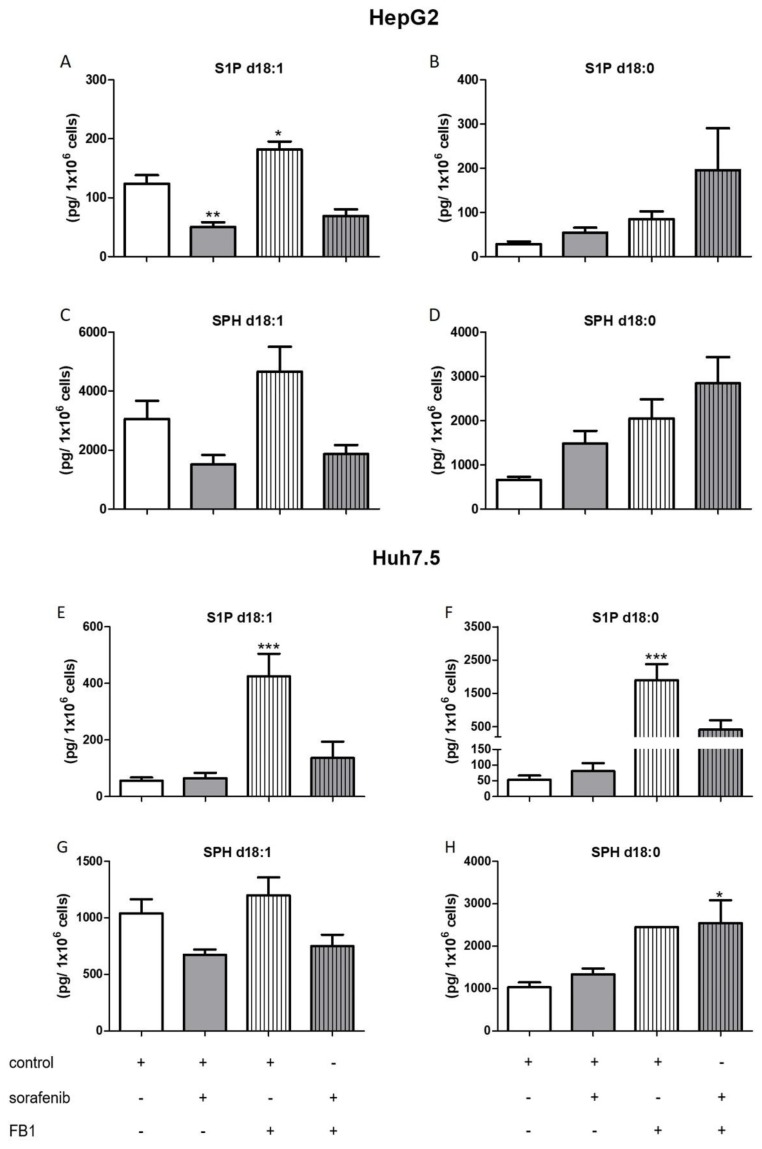
Influence of sorafenib and FB1 on concentrations of S1P and sphingosine in HepG2 and Huh7.5 cells. The lipids were measured by LC-MS/MS. The cells (HepG2 in (**A**)–(**D**) and Huh7.5 in (**E**)–(**H**)) were treated with or without 10 µM SKI II for 2 h before the addition of vehicle (control; 0.2% DMSO) or 5 µM sorafenib and further incubation for 24 h. All results are presented as means ± SEM of three independent experiments. * *p* < 0.05, ** *p* < 0.01, *** p< 0.001 compared to control in one-way ANOVA followed by Bonferroni post-tests. For SPH d18:0 in Huh7.5 cells treated with FB1, only one experiment could be evaluated.

**Figure 8:**
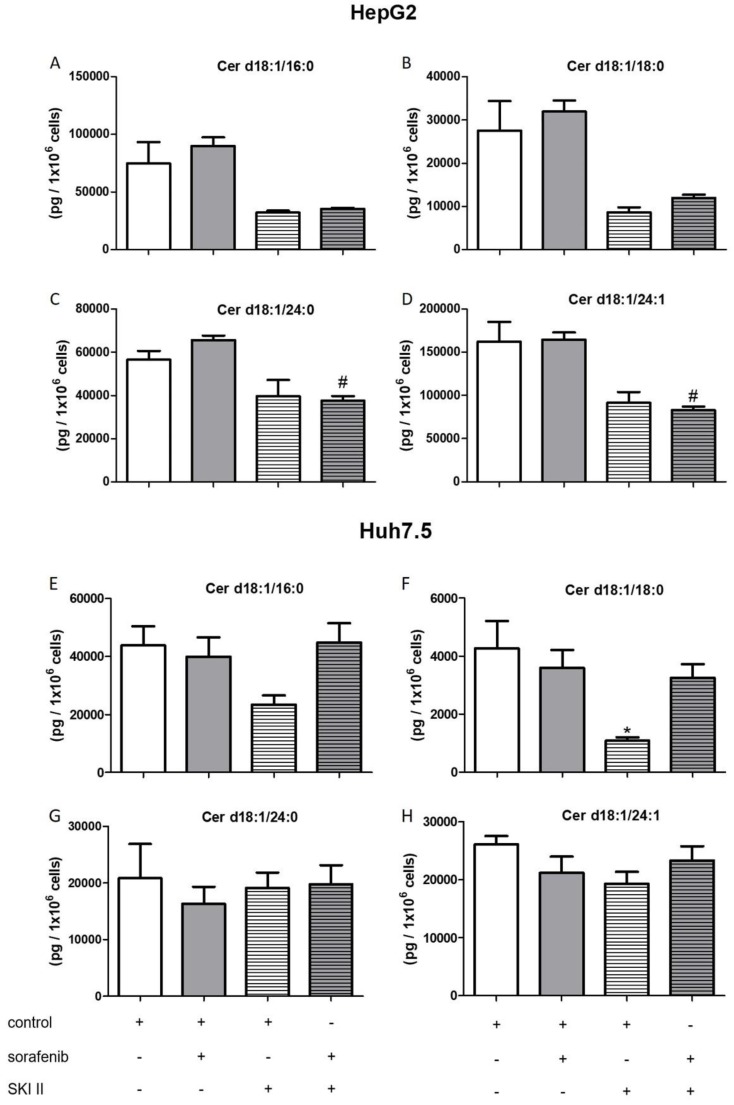
Influence of sorafenib and SKI II on concentrations of d18:1 ceramides in HepG2 and Huh7.5 cells. The lipids were measured by LC-MS/MS. The cells (HepG2 in (**A**)–(**D**) and Huh7.5 in (**E**)–(**H**)) were treated with or without 25 µM FB1 for 1 h before the addition of vehicle (control; 0.2% DMSO) or 5 µM sorafenib and further incubation for 24 h. All results are presented as means ± SEM of 3–6 independent experiments. * *p* < 0.05 compared to control; ^#^ *p* < 0.05 compared to sorafenib in one-way ANOVA followed by Bonferroni post-tests.

**Figure 9:**
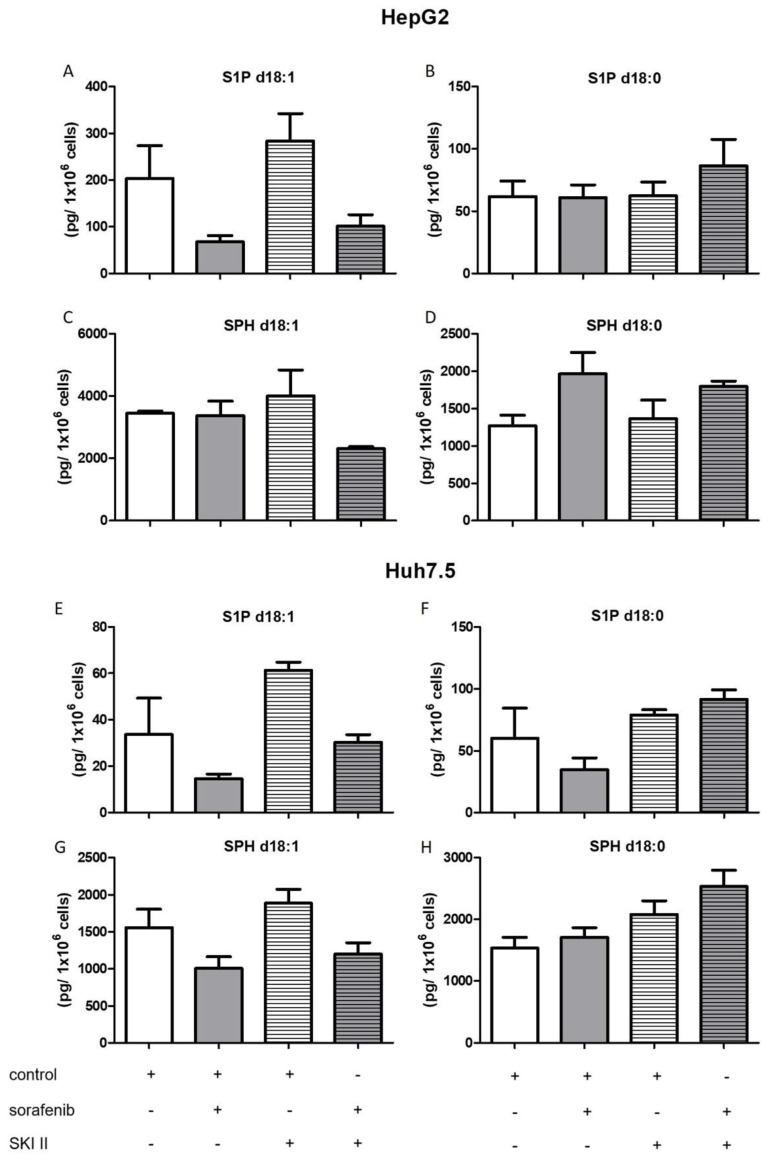
Influence of sorafenib and SKI II on concentrations of S1P and sphingosine in HepG2 and Huh7.5 cells. The lipids were measured by LC-MS/MS. The cells (HepG2 in (**A**)–(**D**) and Huh7.5 in (**E**)–(**H**)) were treated with or without 10 µM SKI II for 2 h before the addition of vehicle (control; 0.2% DMSO) or 5 µM sorafenib and further incubation for 24 h. All results are presented as means ± SEM of three independent experiments.

The influence of the aforementioned treatments on concentrations of ceramides with a double bond (d18:1), sphingosine, and S1P are summarized in Figure 6, Figure 7, Figure 8 and Figure 9. FB1 did not alter d18:1 ceramide levels in HepG2 cells. In contrast, FB1 significantly decreased all d18:1 ceramide concentrations except for Cer d18:1/24:0 in Huh7.5 cells (Figure 6). Interestingly, FB1 significantly increased S1P concentrations in both cell lines, suggesting that it had an inhibitory effect on the conversion of S1P via sphingosine to ceramides, which is in agreement with FB1’s function as a ceramide synthase inhibitor (Figure 7). The inhibitors SLP and SLM did not have a significant effect on the ceramide levels of HepG2 and Huh7.5 cells, neither alone nor in combination with sorafenib (Table S2).

### 2.4. Influence of NAC on the Formation of Dihydroceramides

As shown in Figure 4 and Figure 5, sorafenib stimulation resulted in an increase in dihydroceramides. Since sorafenib is known to regulate oxidative stress, we therefore intended to investigate to which extent these effects were caused by sorafenib-induced ROS (reactive oxygen species) production. The cells were pre-stimulated with different concentrations of the known antioxidant N-acetyl-cysteine (NAC) 1 h before 5 µM sorafenib was added for another 24 h.

**Figure 10:**
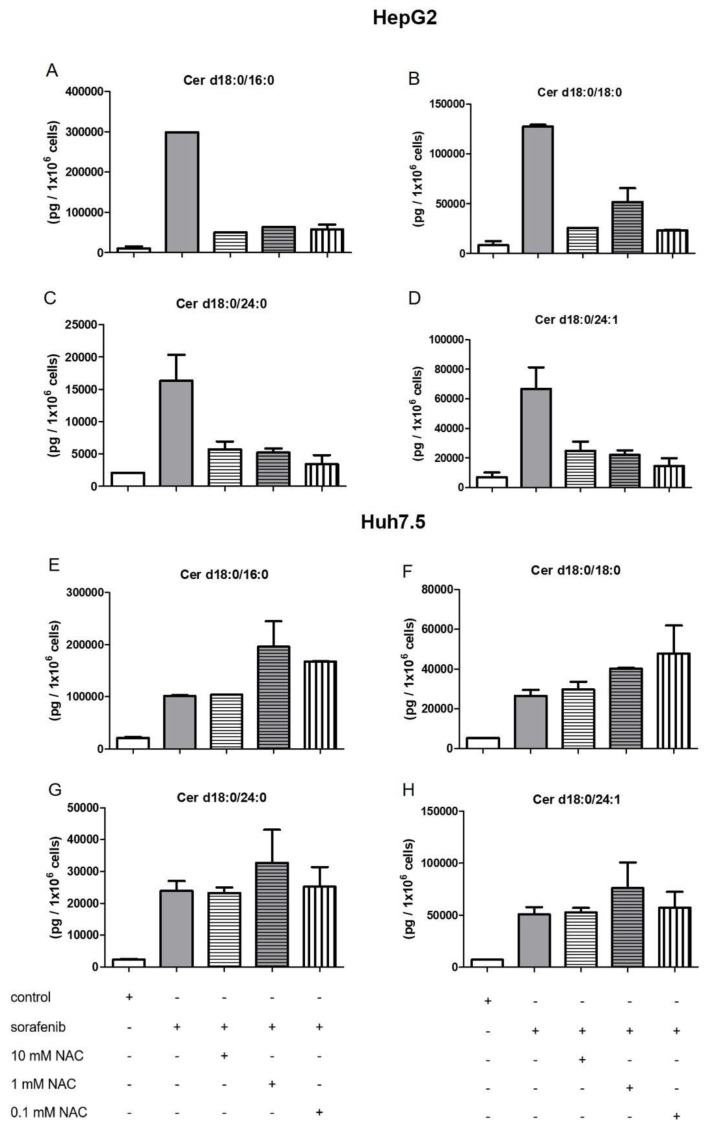
Influence of N-acetyl-cysteine (NAC) on sorafenib-mediated dihydroceramide accumulation of HepG2 and Huh7.5 cells. The lipids were measured by LC-MS/MS. The cells (HepG2 in (**A**)–(**D**) and Huh7.5 in (**E**)–(**H**)) were treated with 10 mM, 1 mM, or 0.1 mM of NAC for 1 h. Data are derived from 1–2 independent experiments performed in triplicate. For d18:0/16:0 in HepG2 cells treated with sorafenib, 10 mM + sorafenib, 0.1 mM + sorafenib. For d18:0/16:0 in Huh7.5 cells treated with 10 mM + sorafenib and d18:0/18:0 treated 10 mM + sorafenib, only one experiment could be evaluated.

As shown in Figure 10, the pretreatment of both cell lines with NAC was not able to affect significantly the concentrations of dihydroceramides.

## 3. Discussion

**Figure 11:**
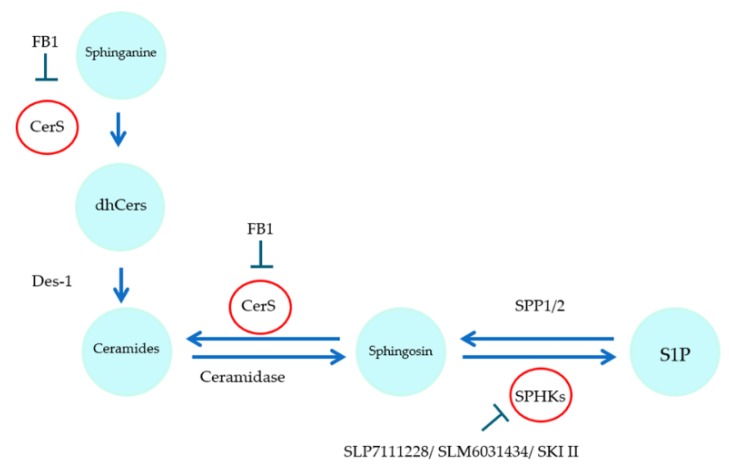
Simplified overview of the target enzymes affected by the inhibitors used.

The sphingolipid pathway plays an important role in cell survival [4], and sphingolipids appear as key metabolites in the regulation of hepatocellular homeostasis [28]. The aim of this study was to explore whether sorafenib, the most frequently applied systemic HCC therapy to date, has an influence on sphingolipid metabolism. Furthermore, it was investigated whether modification of the sphingolipid pathway affects the proliferation and viability of HCC in vitro (Figure 11). In addition, the influence of sorafenib in combination with the specific SPHK inhibitors SLP and SLM was not investigated before. The present data demonstrate that stimulation of HCC cell lines with sorafenib induces an upregulation of dihydroceramides, yet the reduction of cell viability is mostly not attributed to the observed alterations of the sphingolipid pathway. Furthermore, stimulation with FB1 and especially SKI II was able to significantly downregulate the proliferation of HCC cell lines. Interestingly, this downregulation occurred without affecting the formation of dihydroceramides.

Previous studies demonstrated that sorafenib is able to modulate various metabolic pathways, particularly regarding sphingolipids. A combination of sorafenib with vorinostat induced a ceramide-dependent promotion of cell death associated with CD95 signaling in various tumor cell lines [20] and an elevation of dihydroceramides was observed upstream of CD95 activation [19]. However, both studies focused on co-stimulation while not investigating on treatment with sorafenib alone.

Since the sphingolipid pathway has already been shown to affect HCC pathophysiology [29], we assumed that the stimulation of HCC cell lines with inhibitors of the sphingolipid pathway may regulate HCC proliferation *in vitro*. In the current study, the ceramide synthase inhibitor FB1, the specific SPHK1 inhibitor SLP7111228, the specific SPHK2 inhibitor SLM6031434, and SKI II, an inhibitor of both SPHKs, were used. There are several studies that have demonstrated that S1P is probably promoting the invasiveness and metastasis of HCC via the action of mainly SPHK1 [30,31]. Furthermore, various studies have shown that an additional inhibition of SPHKs can support sorafenib treatment. This could be shown not only in HCC cell lines [15], but also in cholangiocell carcinoma cell lines [32], pancreatic adenocarcinoma (Bxpc-3), and renal carcinoma (A-498) cells [33].

In the current study, we could show that FB1 and especially SKI II were able to reduce the proliferation of HepG2 and Huh7.5 cells more effectively than sorafenib (Figure 1 and Figure 2). Therefore, we investigated whether these effects are due to an increased induction of apoptosis or necrosis. As shown in the current study, sorafenib is able to induce apoptosis in HepG2 cells as well as in Huh7.5 cells (Figure 3A,C). This is in line with the results of further studies [34,35,36]. Interestingly, neither FB1 nor SKI II induced increased apoptosis or necrosis in both cell lines (Figure 3A–D). However, data from further studies have shown that FB1 is able to induce apoptosis in different cell lines. [37] were able to prove this to IHKE cells (proximal tubule cells). Furthermore, it could be shown that the stimulation of SNO cells with 1.25 µM FB1 led to the induction of apoptosis [38]. Stimulation with FB1 also led to increased apoptosis in Ges-1 cells [39]. Moreover, SKI II was also shown to induce apoptosis in HL-60 and U937 cells [40], in RNK-16 and NKL cells [41], and in HeLa and SiHa cells as well [42]. Since our current data are contradictory to the above-mentioned results, we assume that the induction of cell cycle arrest by FB1 and SKI II could resemble a possible explanation for the observed controversy. [43] demonstrated that stimulation with 5 µM FB1 was already sufficient to induce cell cycle arrest in CV-1 cells. Furthermore, [44] could show that the administration of FB1 in SD rats led to changes in the expression of genes associated with the cell cycle. Stimulation of the rat brain glioma cell line C6 with FB1 also led to cell cycle arrest. Interestingly, this was observed at concentrations that were not able to influence cell viability [45]. In the current study, we observed both a reduction of proliferation (Figure 1) as well as of cell viability (Figure S1), thus assuming that cell cycle arrest in HepG2 and Huh7.5 cells is possible. Other authors further implied that FB1 significantly influences the expression of cytochrome P450, which in turn influences important functional pathways of HCC in vitro [46]. Yet, since FB1 is a mycotoxin with a well-known promoting role in HCC in vivo [23], further data are needed in order to decipher its mechanistic role in HCC pathogenesis.

SKI II is also capable of inducing cell cycle arrest. Stimulation of SGC7901 cells with SKI II led to cell cycle arrest [47]. It was also shown that SKI II is able to induce cell cycle arrest in A498 cells [48]. Stimulation of HepG2 cells with 5 µM SKI II could additionally increase cell sensitivity to 5-fluorouracil and co-administration of 5-fluorouracil and SKI II led to a reduced expression of sirtuin-1, phosphorylated insulin-like growth factor 1 receptor β, and osteopontin [16]. These proteins are known to affect cell growth, chemoresistance, metastasis, and invasion of cancer cells [49,50,51,52,53]. Further studies showed a reduced ß-catenin expression, leading to a reduction of the proto-oncogenes c-Myc and cyclin D1 in HCC cells upon stimulation with SKI II [14]. In this context, sphingolipid-independent effects of SKI II on HCC cell lines should also be considered in the interpretation of the significant downregulation of HCC proliferation upon stimulation with SKI II, as observed in our study (Figure 1 and Figure 2).

However, sorafenib itself is also able to substantially affect the cell cycle. It could be shown that sorafenib had a different effect on the cell cycle of distinct tumor cell lines. It led to a cell cycle arrest in PC3, Hela, Calu6, and U205 cells, while it did not affect the cell cycle in Bax-/-HCT116, HT29, SKOV3, and H460 cells [54]. Furthermore, it is possible that the effects of SKI II on proliferation observed by us in the current study were mainly independent from sphingolipids and merely induced by alternative pathways such as possibly due to the inhibition of sirtuin-1, phosphorylated insulin-like growth factor 1 receptor ß, and osteopontin, as described by [16]. Certainly, further data are mandatory in order to decipher the underlying mechanism.

In this work, we demonstrate that sorafenib induces a significant accumulation of dihydroceramides (Figure 4 and Figure 5). It is already known that sorafenib affects oxidative stress, as already mentioned. In particular, [55] showed that the induction of oxidative stress is able to inhibit dihydroceramide desaturase, which in turn leads to increased dihydroceramide levels. In this work, we demonstrated a non-significant trend of NAC, reducing dihydroceramide accumulation in HepG2 cells. In Huh7.5 cells, stimulation with NAC does not seem to have any effect on dihydroceramide accumulation. Despite the fact that in our study, NAC did not express any significant effects in the levels of dihydroceramides, we assume that the investigation of further ROS-abrogators may unravel the association of dihydroceramide levels and oxidative stress. Regarding FB1, already published data show that FB1 is also capable of inducing oxidative stress. This has been already demonstrated in vitro [56] as well as in vivo [57]. [22] could prove that FB1 promotes an antioxidative response via NrF2 in HepG2 cells. This could possibly explain why FB1 stimulation alone did not lead to the accumulation of dihydroceramides.

Furthermore, co-treatment of Huh7.5 cells with sorafenib and SLP, respectively SLM, led to a decrease in S1P such that the values could not be detected anymore by LC/MS-MS (Table S3) while not affecting the proliferation of Huh 7.5 cells (Figure 2). This leads to the assumption that the aforementioned results [15,32,33] may potentially not be caused by an effect on SPHK2. Furthermore, considering the limited effect of SLP and SLM on proliferation, also S1P appears not to be essential for the survival of the cells. However, we cannot exclude changes of S1P levels in specific cell compartments, since we only measured the S1P content in whole cells.

In addition, in our current results, we observed that the modification of the sphingolipid metabolism differs substantially within the investigated cell lines. This is in line with the well-known significant heterogeneity of HCC [58]. A further study could show that the majority of HCC tissues shows a remarkable intratumoral heterogeneity [8], which is postulated as a possible explanation for the variable responsiveness of patients to sorafenib treatment [59]. Despite these limitations, according to our results, SKI II may constitute a promising novel compound in systemic HCC treatment strategies.

In summary, we could show that sorafenib exerts antiproliferative effects and enhances dihydroceramides levels in HCC *in vitro*. However, the inhibition of various enzymatic steps of the sphingolipid metabolism neither abrogated nor potentiated the effects of sorafenib. This implies that its antiproliferative effect is not associated to alterations in sphingolipid contents and involves further metabolic pathways. Additionally, the observed antiproliferative effects of FB1 and SKI II encourage further investigations on the role of these drugs in order to investigate their potential as novel HCC therapeutics.

## 4. Materials and Methods

### 4.1. Materials

Sorafenib was purchased from LC Laboratories (Woburn, MA, USA). SLP7111228 (SLP), SLM6031434 (SLM), SKI II, and staurosporine were purchased from Merck (Darmstadt, Germany). FB1 was purchased from Enzo Life Sciences (Farmingdale, NY, USA). All chemicals were dissolved in DMSO. N-Acetyl-L-cysteine was purchased from Sigma-Aldrich (Taufkirchen, Germany)

### 4.2. Cell Culture

The human HCC cell lines Huh7.5 and HepG2 were cultured in DMEM GlutaMax medium (Dulbecco’s Modified Eagle Medium, Life Technologies, Darmstadt, Germany) with 10% FCS (fetal calf serum, Biochrom AG, Berlin, Germany) and 1% Pen/Strep (Life Technologies, Darmstadt, Germany).

### 4.3. Proliferation Assay

A total of 2 × 10^4^ Huh7.5 and HepG2 cells per well were placed in a 24-well plate in medium with 0.2 µCi/mL [^3^H] methyl-thymidine. The cells were stimulated as described for 24 h, 48 h, and 72 h. After completion of stimulation, the medium was removed, and the cells were washed twice with ice-cold PBS (Dulbecco´s Phosphate-buffered Saline, -Ca^2+^, -Mg^2+^, Life Technologies, Darmstadt, Germany). Subsequently, cells were incubated for 30 min with an ice-cold 5% (*w*/*v*) trichloroacetic acid (TCA) and washed twice with 5% TCA. To dissolve the DNA, cells were finally incubated with 1 M NaOH for 30 min at 37 °C and then transferred to a scintillation vial. The solution was neutralized by adding 250 µL glacial acetic acid. Finally, 3 mL of the scintillation mixture (Irgasafe Plus, PerkinElmer Inc, Waltham, MA, USA) was added. The radioactivity was measured with a β-counter (Tri Carb 2100 TR, GMI, Ramsey, USA).

### 4.4. DNA Fragmentation ELISA

First, 5 × 10^3^ Huh7.5 and respectively 3 × 10^3^ HepG2 cells were plated in a 96-well plate and stimulated in starvation medium as indicated for 24 h. The cells or the supernatant were directly taken for the detection of DNA fragmentation (Cell Death Detection ELISA PLUS, Roche Diagnostics GmbH, Mannheim, Germany) according to the manufacturer’s protocol.

### 4.5. Cell Viability

First, 15 × 10^3^ and 20 × 10^3^ Huh7.5 and HepG2 cells per well were placed in a cell culture microplate, 96-well, PS, F-bottom, µClear^®^, black, CELLSTAR^®^ (Greiner Bio-One, Frickenhausen, Germany) and stimulated as described in 130 µl growth medium. After 24 h of stimulation, 20 µL AlamarBlue™ Cell Viability Reagent (Thermo Fisher Scientific, Hampton, NH, USA) was added to the cells and the medium controls. After 4 h of incubation, the fluorescence was measured at an absorbance of 540 nm and an emission of 590 nm in a microplate reader SpectraMax M5 (Molecular Devices, San Jose, CA, USA).

### 4.6. Liquid Chromatography Tandem Mass Spectrometry

Prior to stimulation, cells were starved for 24 h in DMEM + 0.1% BSA. After stimulation for the indicated time points, cells were subjected to LC-MS/MS.

The sphingolipids were quantified by the Institute of Clinical Pharmacology under the direction of Prof. Dr. med. Dr. rer.nat. Gerd Geißlinger using high-performance liquid chromatography tandem mass spectrometry (LC-MS/MS). All reference substances and internal standards were obtained from Avanti Polar Lipids, Alabaster, USA.

For the analysis of the sphingoid bases SPH d18:1, SPH d18:0; SPH d20:1, SPH d20:0, S1P d18:1, and S1P d18:0, 10^6^ cells were used. Cell pellets were resuspended with 200 µL extraction buffer (citric acid 30 mM, disodium hydrogen phosphate 40 mM) and spiked with 20 µL of the internal standard mixture (SPH d18:1-d7, SPH d18:0-d7, S1P d18:1-d7, and S1P d18:0-d7 in methanol).

Samples were mixed with 600 µl methanol:chloroform:HCl (15:83:2, *v*/*v*/*v*) and vortex-mixed for 60 s. After centrifugation (5 min, ca. 15,000 g), the lower organic phase was evaporated at 45 °C under a gentle stream of nitrogen and reconstituted in 100 µL methanol-formic acid (95:5, *v*/*v*). For the preparation of calibration standards and quality control samples, 20 µL of a working solution were processed as stated instead of the cell pellet. Quality control samples of three different concentration levels (low, middle, high) were run as the initial and final samples of each run.

An Agilent 1290 Infinity UHPLC system equipped with a Zorbax Eclipse Plus C8 UHPLC column (30 mm × 2.1 mm ID, 1.8 μm, 100 Å; Agilent Technologies, Waldbronn, Germany) was used for chromatographic separation. The HPLC mobile phases were water:formic acid (99.5:0.5, *v*/*v*) (A) and acetonitrile:isopropyl alcohol:acetone:formic acid (50:30:19:1, *v*/*v*/*v*/*v*) (B). The initial buffer composition of 55% (A)/45% (B) was held for 30 s, and then within 1 min, it linearly changed to 0% (A)/100% (B). After holding 100% (B) for 1.5 min, the composition was linearly changed within 6 s to 55% (A)/45% (B), and the column was re-equilibrated with the initial conditions. The running time for every sample (injection volume of 5 μL) was 4.5 min. After every sample injection, a methanol sample was run to avoid carry-over effects.

The MS/MS analyses were performed using a triple quadrupole mass spectrometer QTRAP5500 (Sciex, Darmstadt, Germany) equipped with a Turbo-V-source operating in positive electrospray ionization (ESI) mode. The analysis was done in Multiple Reaction Monitoring (MRM) mode, recording two precursor-to-product ion transitions per analyte, each with a dwell time of 20 ms. Data acquisition was done using Analyst Software V 1.6.3, and quantification was performed with MultiQuant Software V 3.0.2 (both Sciex, Darmstadt, Germany), employing the internal standard method (isotope dilution mass spectrometry). Variations in accuracy were less than 15% over the whole range of calibration, except for the lower limit of quantification, where a variation in accuracy of 20% was accepted.

The analysis of sphingoid bases and ceramides was performed as described previously [60]. In brief, cell pellets of 10^5^ cells were resuspended with 200 µL of extraction buffer (citric acid 30 mM, disodium hydrogen phosphate 40 mM) and spiked with 20 µL of the internal standard before liquid–liquid extraction. After evaporation at 45 °C under a gentle stream of nitrogen, samples were reconstituted in 100 µl tetrahydrofuran/water 9:1 (*v*/*v*) with 0.2% formic acid and 10 mM ammonium formate.

For chromatographic separation, an Agilent 1290 Infinity UHPLC system equipped with a Zorbax Eclipse Plus C18 UHPLC column (50 mm × 2.1 mm ID, 1.8 μm, 100 Å; Agilent Technologies, Waldbronn, Germany) was used. The mobile phases used for the gradient separation of the analytes were water with 0.2% formic acid and 10 mM ammonium formate (A) and acetonitrile:isopropyl alcohol:acetone:formic acid (50:30:19.8:0.2, *v*/*v*/*v*/*v*) (B).

MS/MS analysis and data acquisition were performed as described previously [60] and as stated above.

### 4.7. Statistical Analysis

The unpaired t-test was used for the statistical analysis of two groups. One-way analysis of variance with subsequent Bonferroni post hoc analysis was used for the comparison of more than two groups. A one-sample t-test was performed after previous normalization. The data are expressed as means ± SEM. All statistics were evaluated with the program GraphPad Prism (version 5; GraphPad Software, San Diego, CA, USA).

## Supplementary Materials

Supplementary materials can be found at https://www.mdpi.com/1422-0067/21/7/2409/s1.

## Abbreviations

DMSO	dimethylsulfoxid
FB1	fumonisin B1
HCC	hepatocellular carcinoma
LC-MS/MS	liquid chromatography/tandem mass spectrometry
NAC	N-acetyl-cysteine
ROS	reactive oxygen species
SPHK1	sphingosine kinase 1
SPHK2	sphingosine kinase 2
SPHKs	sphingosine kinases
SLP	SLP7111228
SLM	SLM6031434
S1P	sphingosine 1-phosphate
TCA	trichloroacetic acid

## References

[1] Torre L.A., Bray F., Siegel R.L., Ferlay J., Lortet-Tieulent J., Jemal A. (2015). Global cancer statistics, 2012. CA Cancer J. Clin..

[2] Forner A., Da Fonseca L.G., Díaz-González Á., Sanduzzi-Zamparelli M., Reig M., Bruix J. (2019). Controversies in the management of hepatocellular carcinoma. JHEP Rep..

[3] Llovet J.M., Ricci S., Mazzaferro V., Hilgard P., Gane E., Blanc J.F., De Oliveira A.C., Santoro A., Raoul J.L., Forner A. (2008). Sorafenib in advanced hepatocellular carcinoma. N. Engl. J. Med..

[4] Newton J., Lima S., Maceyka M., Spiegel S. (2015). Revisiting the sphingolipid rheostat: Evolving concepts in cancer therapy. Exp. Cell Res..

[5] Ogretmen B. (2018). Sphingolipid metabolism in cancer signalling and therapy. Nat. Rev. Cancer.

[6] Ruangsiriluk W., Grosskurth S.E., Ziemek D., Kuhn M., des Etages S.G., Francone O.L. (2012). Silencing of enzymes involved in ceramide biosynthesis causes distinct global alterations of lipid homeostasis and gene expression. J. Lipid Res..

[7] Marí M., Fernández-Checa J.C. (2007). Sphingolipid signalling and liver diseases. Liver Int..

[8] Friemel J., Rechsteiner M., Frick L., Böhm F., Struckmann K., Egger M., Moch H., Heikenwalder M., Weber A. (2015). Intratumor heterogeneity in hepatocellular carcinoma. Clin. Cancer Res..

[9] Li G., Liu D., Kimchi E.T., Kaifi J.T., Qi X., Manjunath Y., Liu X., Deering T., Avella D.M., Fox T. (2018). Nanoliposome C6-Ceramide Increases the Anti-tumor Immune Response and Slows Growth of Liver Tumors in Mice. Gastroenterology.

[10] Senkal C.E., Ponnusamy S., Bielawski J., Hannun Y.A., Ogretmen B. (2010). Antiapoptotic roles of ceramide-synthase-6-generated C16-ceramide via selective regulation of the ATF6/CHOP arm of ER-stress-response pathways. FASEB J..

[11] Lu P.H., Chen M.B., Liu Y.Y., Wu M.H., Li W.T., Wei M.X., Liu C.Y., Qin S.K. (2017). Identification of sphingosine kinase 1 (SphK1) as a primary target of icaritin in hepatocellular carcinoma cells. Oncotarget.

[12] Pewzner-Jung Y., Brenner O., Braun S., Laviad E.L., Ben-Dor S., Feldmesser E., Horn-Saban S., Amann-Zalcenstein D., Raanan C., Berkutzki T. (2010). A critical role for ceramide synthase 2 in liver homeostasis: II. Insights into molecular changes leading to hepatopathy. J. Biol. Chem..

[13] French K.J., Upson J.J., Keller S.N., Zhuang Y., Yun J.K., Smith C.D. (2006). Antitumor activity of sphingosine kinase inhibitors. J. Pharmacol. Exp. Ther..

[14] Liu H., Zhang C.X., Ma Y., He H.W., Wang J.P., Shao R.G. (2016). SphK1 inhibitor SKI II inhibits the proliferation of human hepatoma HepG2 cells via the Wnt5A/β-catenin signaling pathway. Life Sci..

[15] Beljanski V., Lewis C.S., Smith C.D. (2011). Antitumor activity of sphingosine kinase 2 inhibitor ABC294640 and sorafenib in hepatocellular carcinoma xenografts. Cancer Biol. Ther..

[16] Grbčić P., Tomljanović I., Klobučar M., Pavelić S.K., Lučin K., Sedić M. (2017). Dual sphingosine kinase inhibitor SKI-II enhances sensitivity to 5-fluorouracil in hepatocellular carcinoma cells via suppression of osteopontin and FAK/IGF-1R signalling. Biochem. Biophys. Res. Commun..

[17] Savić R., He X., Fiel I., Schuchman E.H. (2013). Recombinant human acid sphingomyelinase as an adjuvant to sorafenib treatment of experimental liver cancer. PLoS ONE.

[18] Yin X., Xiao Y., Han L., Zhang B., Wang T., Su Z., Zhang N. (2018). Ceramide-Fabricated Co-Loaded Liposomes for the Synergistic Treatment of Hepatocellular Carcinoma. AAPS PharmSciTech.

[19] Park M.A., Mitchell C., Zhang G., Yacoub A., Allegood J., Häussinger D., Reinehr R., Larner A., Spiegel S., Fisher P.B. (2010). Vorinostat and sorafenib increase CD95 activation in gastrointestinal tumor cells through a Ca^2+^-de novo ceramide-PP2A-reactive oxygen species-dependent signaling pathway. Cancer Res..

[20] Park M.A., Zhang G., Martin A.P., Hamed H., Mitchell C., Hylemon P.B., Graf M., Rahmani M., Ryan K., Liu X. (2008). Vorinostat and sorafenib increase ER stress, autophagy and apoptosis via ceramide-dependent CD95 and PERK activation. Cancer Biol. Ther..

[21] Stefanovic M., Tutusaus A., Martinez-Nieto G.A., Bárcena C., de Gregorio E., Moutinho C., Barbero-Camps E., Villanueva A., Colell A., Marí M. (2016). Targeting glucosylceramide synthase upregulation reverts sorafenib resistance in experimental hepatocellular carcinoma. Oncotarget.

[22] Arumugam T., Pillay Y., Ghazi T., Nagiah S., Abdul N.S., Chuturgoon A.A. (2019). Fumonisin B1-induced oxidative stress triggers Nrf2-mediated antioxidant response in human hepatocellular carcinoma (HepG2) cells. Mycotoxin Res..

[23] Xue K.S., Tang L., Cai Q., Shen Y., Su J., Wang J.S. (2015). Mitigation of Fumonisin Biomarkers by Green Tea Polyphenols in a High-Risk Population of Hepatocellular Carcinoma. Sci. Rep..

[24] Grammatikos G., Mühle C., Ferreiros N., Schroeter S., Bogdanou D., Schwalm S., Hintereder G., Kornhuber J., Zeuzem S., Sarrazin C. (2014). Serum acid sphingomyelinase is upregulated in chronic hepatitis C infection and non alcoholic fatty liver disease. Biochim. Biophys. Acta.

[25] Grammatikos G., Schoell N., Ferreirós N., Bon D., Herrmann E., Farnik H., Köberle V., Piiper A., Zeuzem S., Kronenberger B. (2016). Serum sphingolipidomic analyses reveal an upregulation of C16-ceramide and sphingosine-1-phosphate in hepatocellular carcinoma. Oncotarget.

[26] Mücke V.T., Gerharz J., Jakobi K., Thomas D., Ferreirós Bouzas N., Mücke M.M., Trötschler S., Weiler N., Welker M.W., Zeuzem S. (2018). Low Serum Levels of (Dihydro-)Ceramides Reflect Liver Graft Dysfunction in a Real-World Cohort of Patients Post Liver Transplantation. Int. J. Mol. Sci..

[27] Saito K., Ikeda M., Kojima Y., Hosoi H., Saito Y., Kondo S. (2018). Lipid profiling of pre-treatment plasma reveals biomarker candidates associated with response rates and hand-foot skin reactions in sorafenib-treated patients. Cancer Chemother. Pharmacol..

[28] Nojima H., Freeman C.M., Gulbins E., Lentsch A.B. (2015). Sphingolipids in liver injury, repair and regeneration. Biol. Chem..

[29] Maceyka M., Rohrbach T., Milstien S., Spiegel S. (2019). Role of Sphingosine Kinase 1 and Sphingosine-1-Phosphate Axis in Hepatocellular Carcinoma. Handb. Exp. Pharmacol..

[30] Bao M., Chen Z., Xu Y., Zhao Y., Zha R., Huang S., Liu L., Chen T., Li J., Tu H. (2012). Sphingosine kinase 1 promotes tumour cell migration and invasion via the S1P/EDG1 axis in hepatocellular carcinoma. Liver Int..

[31] Liu H., Ma Y., He H.W., Zhao W.L., Shao R.G. (2017). SPHK1 (sphingosine kinase 1) induces epithelial-mesenchymal transition by promoting the autophagy-linked lysosomal degradationn of CDH1/E-cadherin in hepatoma cells. Autophagy.

[32] Ding X., Chaiteerakij R., Moser C.D., Shaleh H., Boakye J., Chen G., Ndzengue A., Li Y., Zhou Y., Huang S. (2016). Antitumor effect of the novel sphingosine kinase 2 inhibitor ABC294640 is enhanced by inhibition of autophagy and by sorafenib in human cholangiocarcinoma cells. Oncotarget.

[33] Beljanski V., Knaak C., Zhuang Y., Smith C.D. (2011). Combined anticancer effects of sphingosine kinase inhibitors and sorafenib. Investig. New Drugs.

[34] Liu L., Cao Y., Chen C., Zhang X., McNabola A., Wilkie D., Wilhelm S., Lynch M., Carter C. (2006). Sorafenib blocks the RAF/MEK/ERK pathway, inhibits tumor angiogenesis, and induces tumor cell apoptosis in hepatocellular carcinoma model PLC/PRF/5. Cancer Res..

[35] Zhang L., Li S., Wang R., Chen C., Ma W., Cai H. (2019). Cytokine augments the sorafenib-induced apoptosis in Huh7 liver cancer cell by inducing mitochondrial fragmentation and activating MAPK-JNK signalling pathway. Biomed. Pharmacother..

[36] Garten A., Grohmann T., Kluckova K., Lavery G.G., Kiess W., Penke M. (2019). Sorafenib-Induced Apoptosis in Hepatocellular Carcinoma Is Reversed by SIRT1. Int. J. Mol. Sci..

[37] Seefelder W. (2003). Induction of apoptosis in cultured human proximal tubule cells by fumonisins and fumonisin metabolites. Toxicol. Appl. Pharmacol..

[38] Khan R.B., Phulukdaree A., Chuturgoon A.A. (2018). Concentration-dependent effect of fumonisin B1 on apoptosis in oesophageal cancer cells. Hum. Exp. Toxicol..

[39] Yu S., Jia B., Yang Y., Liu N., Wu A. (2020). Involvement of PERK-CHOP pathway in fumonisin B1-induced cytotoxicity in human gastric epithelial cells. Food Chem. Toxicol..

[40] Yang L., Weng W., Sun Z.X., Fu X.J., Ma J., Zhuang W.F. (2015). SphK1 inhibitor II (SKI-II) inhibits acute myelogenous leukemia cell growth in vitro and in vivo. Biochem. Biophys. Res. Commun..

[41] LeBlanc F.R., Liu X., Hengst J., Fox T., Calvert V., Petricoin E.F., Yun J., Feith D.J., Loughran T.P. (2015). Sphingosine kinase inhibitors decrease viability and induce cell death in natural killer-large granular lymphocyte leukemia. Cancer Biol. Ther..

[42] Kim H.S., Yoon G., Ryu J.Y., Cho Y.J., Choi J.J., Lee Y.Y., Kim T.J., Choi C.H., Song S.Y., Kim B.G. (2015). Sphingosine kinase 1 is a reliable prognostic factor and a novel therapeutic target for uterine cervical cancer. Oncotarget.

[43] Ciacci-Zanella J.R., Merrill A.H., Wang E., Jones C. (1998). Characterization of Cell-cycle Arrest by Fumonisin B1 in CV-1 Cells. Food Chem. Toxicol..

[44] Bondy G.S., Barker M.G., Lombaert G.A., Armstrong C.L., Fernie S.M., Gurofsky S., Huzel V., Savard M.E., Curran I.H. (2000). A comparison of clinical, histopathological and cell-cycle markers in rats receiving the fungal toxins fumonisin B1 or fumonisin B2 by intraperitoneal injection. Food Chem. Toxicol..

[45] Mobio T.A., Anane R., Baudrimont I., Carratú M.R., Shier T.W., Dano S.D., Ueno Y., Creppy E.E. (2000). Epigenetic properties of fumonisin B1: Cell cycle arrest and DNA base modification in C6 glioma cells. Toxicol. Appl. Pharmacol..

[46] Mary V.S., Arias S.L., Otaiza S.N., Velez P.A., Rubinstein H.R., Theumer M.G. (2017). The aflatoxin B1 -fumonisin B1 toxicity in BRL-3A hepatocytes is associated to induction of cytochrome P450 activity and arachidonic acid metabolism. Environ. Toxicol..

[47] Li P.H., Wu J.X., Zheng J.N., Pei D.S. (2014). A sphingosine kinase-1 inhibitor, SKI-II, induces growth inhibition and apoptosis in human gastric cancer cells. Asian Pac. J. Cancer Prev..

[48] Gao P., Peterson Y.K., Smith R.A., Smith C.D. (2012). Characterization of isoenzyme-selective inhibitors of human sphingosine kinases. PLoS ONE.

[49] Chen J., Zhang B., Wong N., Lo A.W., To K.F., Chan A.W., Ng M.H., Ho C.Y., Cheng S.H., Lai P.B. (2011). Sirtuin 1 is upregulated in a subset of hepatocellular carcinomas where it is essential for telomere maintenance and tumor cell growth. Cancer Res..

[50] Choi H.N., Bae J.S., Jamiyandorj U., Noh S.J., Park H.S., Jang K.Y., Chung M.J., Kang M.J., Lee D.G., Moon W.S. (2011). Expression and role of SIRT1 in hepatocellular carcinoma. Oncol. Rep..

[51] Tian X., Hao K., Qin C., Xie K., Xie X., Yang Y. (2013). Insulin-like growth factor 1 receptor promotes the growth and chemoresistance of pancreatic cancer. Dig. Dis. Sci..

[52] Zhao J., Dong L., Lu B., Wu G., Xu D., Chen J., Li K., Tong X., Dai J., Yao S. (2008). Down-regulation of osteopontin suppresses growth and metastasis of hepatocellular carcinoma via induction of apoptosis. Gastroenterology.

[53] Lin F., Li Y., Cao J., Fan S., Wen J., Zhu G., Du H., Liang Y. (2011). Overexpression of osteopontin in hepatocellular carcinoma and its relationships with metastasis, invasion of tumor cells. Mol. Biol. Rep..

[54] Plastaras J.P., Kim S.H., Liu Y.Y., Dicker D.T., Dorsey J.F., McDonough J., Cerniglia G., Rajendran R.R., Gupta A., Rustgi A.K. (2007). Cell cycle dependent and schedule-dependent antitumor effects of sorafenib combined with radiation. Cancer Res..

[55] Idkowiak-Baldys J., Apraiz A., Li L., Rahmaniyan M., Clarke C.J., Kraveka J.M., Asumendi A., Hannun Y.A. (2010). Dihydroceramide desaturase activity is modulated by oxidative stress. Biochem. J..

[56] Yuan Q., Jiang Y., Fan Y., Ma Y., Lei H., Su J. (2019). Fumonisin B1 Induces Oxidative Stress and Breaks Barrier Functions in Pig Iliac Endothelium Cells. Toxins.

[57] Kim S.H., Singh M.P., Sharma C., Kang S.C. (2018). Fumonisin B1 actuates oxidative stress-associated colonic damage via apoptosis and autophagy activation in murine model. J. Biochem. Mol. Toxicol..

[58] Xue R., Li R., Guo H., Guo L., Su Z., Ni X., Qi L., Zhang T., Li Q., Zhang Z. (2016). Variable Intra-Tumor Genomic Heterogeneity of Multiple Lesions in Patients with Hepatocellular Carcinoma. Gastroenterology.

[59] Xu L.X., He M.H., Dai Z.H., Yu J., Wang J.G., Li X.C., Jiang B.B., Ke Z.F., Su T.H., Peng Z.W. (2019). Genomic and transcriptional heterogeneity of multifocal hepatocellular carcinoma. Ann. Oncol..

[60] Gurke R., Etyemez S., Prvulovic D., Thomas D., Fleck S.C., Reif A., Geisslinger G., Lötsch J. (2019). A Data Science-Based Analysis Points at Distinct Patterns of Lipid Mediator Plasma Concentrations in Patients with Dementia. Front. Psychiatr..

