# Supplementary material for: Sorafenib Treatment and Modulation of the Sphingolipid Pathway Affect Proliferation and Viability of Hepatocellular Carcinoma In Vitro
Source: Int J Mol Sci. 2020 Mar 31;21(7):2409. doi: 10.3390/ijms21072409 (PMC7177910; doi:10.3390/ijms21072409)
Supplement: Supplementary file 1 [file ijms-21-02409-s001.pdf]

Effects of sorafenib, FB1 and SPHK inhibitors on cell viability

Cell viability was determined by measuring the capacity of the cells to reduce resazurin to resorufin (AlamarBlue™ assay). Similar to the cell proliferation assay, HepG2 and Huh7.5 cells were pretreated for 1 h with FB1, or for 2 h with SPHK inhibitors, before addition of vehicle or 5 µM sorafenib. As shown in Figure 3, sorafenib significantly reduced viability of both HepG2 and Huh7.5 cells by about 20 and 13 %. Also, FB1 reduced viability of both cell lines to a small but significant extent (Figure 3). Interestingly, SKI-II had no significant effect on viability of both HepG2 and Huh7.5 cells (Figure 3), despite its strong inhibition on cell proliferation already after 24 h in HebG2 cells as shown in Figure 1. Finally, none of the selective SPHK1 and SPHK2 inhibitors had an influence on cell viability (Figure 3A, B).

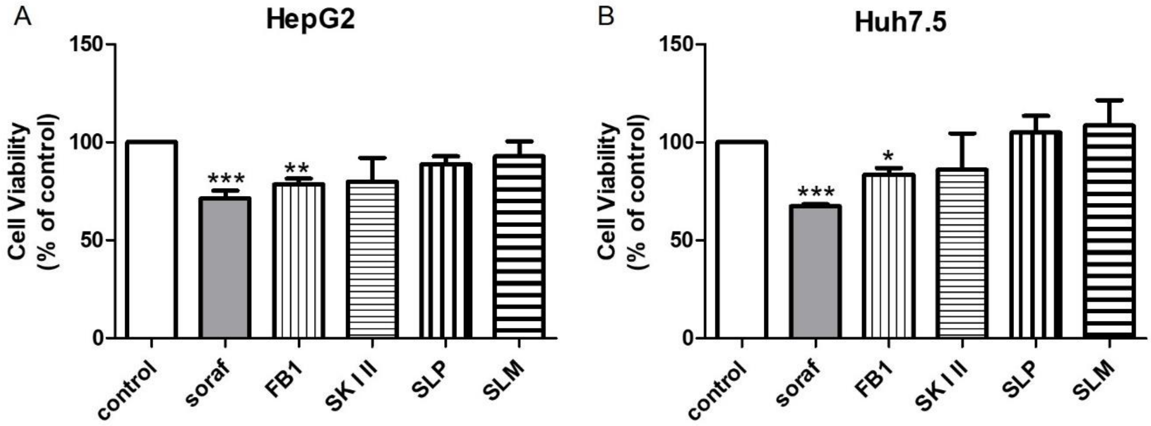

Figure S1. Influence of sorafenib, FB1 and SPHKs inhibitors on viability of HepG2 and Huh7.5 cells. Treatment was performed with vehicle (0.2 % DMSO), 5 µM sorafenib (soraf), 25 µM FB1, 10 µM SKI II, 1 µM SLP7111228 (SLP), or 1 µM SLM6031434 (SLM) for 24 h. All data are derived at least from 3 independent experiments performed in triplicates. All results are presented as means ± SEM; control and sorafenib, n = 12 each; FB1 and SLP, n=4 each; SKI II and SLM, n = 3 each. *P < 0.05, **P < 0.01, ***P < 0.001 in One-sample t-test.

Influence of sorafenib, SLP7111228and SLM6031434*inhibitors on levels of bioactive sphingolipids*

Treatment of the two cell lines with the inhibitors, SLM and SLP, had no significant effects on concentrations of d18:0 and d18:1 ceramides, sphingosine or S1P (Table S1 and S2). Combined treatment with sorafenib and SLP or SLM resulted in undetectable levels of S1P in Huh7.5 cells (Table S3).

Table S1. Influence of sorafenib and SPHK inhibitors on concentrations of d18:0 ceramides in HepG2 and Huh7.5 cells.[1]

**HepG2**	**Cer18:0/16:0**			**Cer18:0/18:0**			**Cer18:0/24:0**			**Cer18:0/24:1** (ng/1x10[6] cells)		
control	9.01	±	2.85	9.12	±	2.81	2.79	±	0.15	13.1	±	2.73
sorafenib	19.4	±	1.93	23.2	±	2.57	9.67	±	0.45	37.1	±	2.13
SLP	8.35	±	2.68	7.06	±	1.99	2.20	±	0.20	9.86	±	1.91
soraf + SLP	19.9	±	3.60	22.3	±	4.99	8.08	±	0.64	32.0	±	1.80
SLM	10.0	±	2.65	10.2	±	2.42	2.82	±	0.30	13.8	±	2.12
soraf + SLM	20.2	±	1.57	24.9	±	2.96	9.81	±	0.73	37.9	±	1.81
**Huh7.5**												
control	12.8	±	3.10	4.03	±	1.12	2.29	±	0.37	7.05	±	1.10
sorafenib	24.0	±	1.76	8.54	±	0.61	5.48	±	1.09	14.2	±	1.41
SLP	8.57	±	1.33	1.35	±	0.32	1.07	±	0.35	2.62	±	0.80

soraf + SLP	36.1	±	3.33	9.44	±	0.41	5.90	±	1.17	15.2	±	3.22
SLM	11.3	±	2.98	3.36	±	0.90	1.36	±	0.07	5.80	±	1.00
soraf + SLM	28.3	±	2.16	8.55	±	0.80	4.26	±	0.68	12.3	±	2.00

[1] For details, see legend of table 3S

Table S2. Influence of sorafenib and SPHK inhibitors on concentrations of d18:1 ceramides in HepG2 and Huh7.5 cells.[1]

**HepG2**	**Cer18:1/16:0**			**Cer18:1/18:0**			**Cer18:1/24:0**			**Cer18:1/24:1** (ng/1x10[6] cells)		
control	74.6	±	18.7	27.5	±	6.89	56.5	±	4.05	162	±	22.8
sorafenib	89.8	±	7.52	31.9	±	2.57	65.6	±	2.07	165	±	8.27
SLP	78.6	±	11.2	25.6	±	5.43	54.0	±	1.22	151	±	10.4
soraf + SLP	107	±	20.7	45.5	±	8.10	72.5	±	13.9	183	±	33.0
SLM	96.9	±	14.9	35.0	±	7.32	70.2	±	4.97	184	±	17.3
soraf + SLM	101	±	11.0	37.3	±	1.59	69.6	±	3.41	180	±	9.16
**Huh7.5**												
control	43.8	±	6.61	4.27	±	0.95	20.8	±	6.01	26.1	±	1.46
sorafenib	39.9	±	6.59	3.59	±	0.63	16.3	±	3.02	21.2	±	2.84
SLP	42.7	±	11.2	2.84	±	1.03	26.6	±	13.8	25.7	±	5.75
soraf + SLP	49.1	±	10.7	3.87	±	0.45	18.1	±	4.62	23.8	±	5.47
SLM	41.7	±	6.22	4.37	±	1.07	19.6	±	3.36	24.9	±	1.39
soraf + SLM	38.7	±	6.20	3.06	±	0.18	15.5	±	2.51	19.7	±	3.07

[1] For details, see legend of table 3S

Table S3. Influence of sorafenib and SPHK inhibitors on concentrations of S1P and sphingosine in HepG2 and Huh7.5 cells.[1]

**HepG2**	**S1P d18:1**			**S1P d18:0**			**SPH d18:1**			**SPH d18:0** (pg/1x10[6] cells)		
control	204	±	70.2	61.8	±	12.4	3446	±	73.2	1267	±	145
sorafenib	67.9	±	13.3	60.8	±	10.3	3374	±	464	1965	±	285
SLP	206	±	72.1	44.5	±	7.07	3713	±	69.9	1266	±	130
soraf + SLP	44.2	±	6.9	27.5	±	5.51	4226	±	659	1892	±	274
SLM	184	±	64.9	43.8	±	7.56	4754	±	177	1616	±	71.2
soraf + SLM	66.4	±	14.9	51.0	±	5.57	3783	±	523	2037	±	223
**Huh7.5**												
control	33.7	±	15.7	60.2	±	24.3	1555	±	247	1533	±	176
sorafenib	14.6	±	2.04	34.8	±	9.43	1006	±	155	1707	±	157
SLP	21.2	±	7.76	21.9	±	5.58	2566	±	655	1856	±	368
soraf + SLP	< LLOQ			< LLOQ			1313	±	298	2011	±	336
SLM	21.6	±	8.69	30.2	±	11.1	1525	±	21.8	1307	±	30.2
soraf + SLM	< LLOQ			19.0	±	2.99	898	±	124	1550	±	161

[1] Concentrations of sphingolipids measured by LC-MS/MS after treatment of HepG2 (1, 3) and Huh7.5 cells (2, 4) with 5 µM sorafenib (soraf) and/or 1 µM SLP7111228 (SLP) and 1 µM SLM6031434 (SLM) for 24 h. All results are presented as means ± SEM (n = 3-6). Concentrations are presented as ng/1x10[6] cells for dihydroceramides and ceramides and as pg/1x10[6] cells for S1P and sphingosine. *P < 0.05, **P < 0.01, ***P < 0.001 compared to control (0.2 % DMSO); [#]P < 0.05, [##]P < 0.01, [###]P < 0.001 compared to sorafenib. <LLOQ, below lower limit of quantification. Control and sorafenib values are the same as in figures 6 -9.
